# Utilizing VIS-NIR Technology to Generate a Quality Index (Q_i_) Model of Barhi Date Fruits at the Khalal Stage Stored in a Controlled Environment

**DOI:** 10.3390/foods13020345

**Published:** 2024-01-22

**Authors:** Abdullah M. Alhamdan

**Affiliations:** Dates Industry and Technology Chair, Department of Agricultural Engineering, College of Food and Agricultural Sciences, King Saud University, Riyadh 12372, Saudi Arabia; alhamdan@ksu.edu.sa

**Keywords:** Barhi dates, Khalal, maturation, quality index, modeling, visible–near-infrared (VIS-NIR), storage

## Abstract

Saudi Arabia is a prominent producer of dates, producing 1.6 million tons annually. There is a need to evaluate the physical properties and quality of fruits non-destructively and then modeled and predict them throughout the storage period. The aim of the current study was to generate a quality index (Q_i_) and visible–near-infrared spectra (VIS-NIR) models non-destructively to predict properties of Barhi dates including objective and sensory evaluations. A total of 1000 Barhi fruits were sorted into three stages of maturation, ranging from 80 to 100% yellowish. The physical properties (hardness, color, TSS, pH, and sensory evaluations) of Barhi dates were measured and modeled with Q_i_ based on VIS-NIR of fresh Barhi fruits and during storage in ambient (25 °C), cold (1 °C), and CA (1 °C with 5%:5% O_2_:CO_2_, 85% RH) conditions for up to 3 months. The prediction of Q_i_ was non-destructively based on VIS-NIR utilizing PLSR and ANN data analysis. The results showed that the shelf-life of stored Barhi fruits were 20, 40, and 120 days corresponding to 25 °C, cold (1 °C), and CA, respectively. It was found that VIS-NIR spectroscopy was helpful in estimating the Q_i_ of Barhi fruits for PLSR and ANN data analysis, respectively, in calibration with an R^2^ of 0.793 and 0.912 and RMSEC of 0.110 and 0.308 and cross-validation with an R^2^ of 0.783 and 0.912 and RMSEC of 0.298 and 0.308. The VIS-NIR spectrum has proven to be an effective method for the evaluation of the Q_i_ of Barhi fruits and their physical properties throughout the supply chain in the handling, processing, transportation, storage and retail sectors. It was found that ANN is more suitable than PLSR analysis.

## 1. Introduction

Saudi Arabia produces more than 14% of the world’s total production of dates, viewing the date palm as the most important fruit tree in country. Barhi dates fruits are tasty, physiologically mature, crisp and firm during the Khalal stage (yellow in color) of maturation [1]. They are also perishable and have a high moisture content of 66% to 75% (wet bases) and water activity 0.95 to 0.97. In this region, Barhi fruits during the Khalal maturation (Bisr) were mainly chosen because of their desirable texture, sweetness, and great flavors [2]. Researchers, food innovators and producers, consumers, and health experts are emphasizing the need for food quality for fruits to be more palatable, healthy, natural, and wholesome.

Fruit losses on farms may reach as high as 25% due to the huge production of Barhi dates over a short season and the lack of commercial means to maintain fresh Barhi at its Khalal maturation stage, which causes a drop in fruit prices during the peak season. Towards the end of the season, Barhi prices gradually rise, reaching as much as ten times the price of that of the average season price [3].

Cooling plays a significant role in decreasing the biological activities, respiration rate and enzymatic activities during storage. Several studies have indicated that any delay in cooling harvested fruits leads to water losses of up to 50% [4]. Etiolation of the fruit surface as well as changes in physical qualities (e.g., weight, density, and color) result from this water loss. Furthermore, moisture losses may lead to an increase in sugar component [5].

Controlled atmospheric (CA) storage is a postharvest preservation technique involving the meticulous regulation of the gaseous composition (oxygen, carbon dioxide, and nitrogen) and environmental conditions (temperature and relative humidity) within enclosed storage facilities. CA storage slows ripening by lowering oxygen levels and increasing carbon dioxide levels, thus extending the shelf-life of various fruits and vegetables. Fruits stored in a low-O_2_ atmosphere slow the ripening, but they continue to ripen when left in the air [6]. Successful CA preservation of Barhi fruits was achieved at a storage temperature of 0 °C where fruits kept fresh for up to 5 months [7] and where best quality was kept within the first three months of storage.

Fruit ripening stages may be assessed using smart agriculture technologies, which might help with quality management. Frequently, methods used to evaluate fruit quality include the titrable acidity (TA), starch content, pH, soluble solid content (SSC), ratio of SSC to TA, tissue stiffness, and physical features including size, shape, color, and appearance [8]. Routine measurements of physicochemical properties of foods to evaluate food quality during the whole chain from harvest to consumers are expensive and time-consuming, and mostly destructive to the samples. It is crucial for commercial management to avoid destructive tests through a quick prediction of food properties during the various stages of processing. 

The food quality index (Q_i_) [9,10] can describe a theoretical/empirical model developed to define product general characteristics. In research and industry applications, objective measurements are favored over sensorial tests. These technologies are more accurate, eliminate arbitrator discrepancies, and provide consumers, businesses, and academics with one standardized language [11]. It is important to track the food quality attributes over time with an effective prediction model such as a Q_i_ to assist food manufacturers and consumer acceptance. After that, additional predictions for the Q_i_ model can be made using non-destructive testing, namely, near-infrared spectroscopy (NIR).

Using near-infrared spectroscopy (NIR) is a frequent non-destructive detection method for rapid assessment of fruit qualities [12]. In several studies, researchers employed near-infrared (NIR) spectroscopy in an attempt to predict the chemical and physical characteristics of many fruits, both in their fresh state and after undergoing processing or storage [13,14,15,16]. Furthermore, spectroscopy and fruit sensory evaluation have been correlated in a number of research studies [15,17,18]. The integration of appropriate statistical analysis with near-infrared (NIR) spectroscopy can be deemed an effective technique for evaluating both quantifiable and qualitative aspects of food quality, including its inherent attributes and sensory characteristics. By establishing a comprehensive Q_i_ model that incorporates VIS-NIR spectral data, the assessment of food quality can be facilitated and optimized across the entire food chain, from initial production through consumption, while simultaneously accounting for potential variations during shelf life.

The wavelength range of 300–2000 nm, which includes VIS-NIR, was used to estimate the soluble sugar concentration in apple fruits with a relatively good R^2^ of 0.91–0.97 [16,19,20]. Cherry fruits were assessed for soluble sugar content utilizing VIS-NIR spectra at a 600–1100 nm wavelength with a standard error of prediction (SEP) of 0.75, which measures the average difference between a set of samples’ predicted and actual values [21]. For kiwi fruits, the physical parameters SSC and Hue angle were conducted using near-infrared estimation with an R^2^ of 0.82 and 0.93, respectively [22]. NIR spectroscopy was used [23] at 900–1700 nm to classify Shahani dates into four maturity phases: Kimiri, Khalal, Rutab, and Tamr, where the R^2^ values for the moisture content and TSS were 0.98 and 0.96, respectively, in the predicted models. According to Gómez [24], mandarin has six broadband peaks on its absorption curve. Spectroscopic analysis performed in the NIR region reveals a significant absorption peak at 672 nm, suggesting the presence of pigments like chlorophyll. The presence of chlorophyll leads to the distinctive green color observed in the fruit.

While PLSR (partial least squares regression) is a classic linear tool in chemometrics, ANNs (artificial neural networks) offer a powerful alternative for modeling complex nonlinear relationships between input and output data. Introduced relatively recently to the field, ANNs are finding diverse applications in chemometrics, including mapping, regression, modeling, clustering, and classification [25]. Notably, their ability to interpret and quantify overlapping peaks and reduce interference effects in mixed spectra makes them particularly valuable for food investigations [26].

There is a need to investigate and quantify both objective and subjective measurements of the fruit quality throughout the storage period of controlled atmospheric (CA) storage. Thus, the objective of this work was to model a Q_i_ for Barhi fruits involving sensory and objective assessments, followed by investigating the possibility of forecasting the Q_i_ using VIS-NIR of those properties non-destructively. This reduces the required money, time, and labor for routine work and sensory evaluation of fresh and stored products throughout the processing and market chain.

## 2. Materials and Methods

### 2.1. Fruit Samples Preparation and Storage

Barhi fruits, identified as having reached the Khalal maturity stage, were harvested from a date palm farm situated within the Riyadh region of Saudi Arabia. Thereafter, on the same day, the aforementioned fruits were transported to the food processing laboratory located at King Saud University. After being cleaned gently from dust with compressed air (3 bar), a total of 1000 Barhi fruits were sorted to three stages of maturation, ranging from 80 to 100% yellowish. A total of 75% of the dataset (750 fruits) was used for model training (calibration and cross-validation) while the remaining 25% (250 fruits) was utilized for testing the model performance. Barhi fruits were first divided into three groups for color (non-destructive test) followed by MC and TSS), texture, and sensory evaluation. Fruits were scanned with Flex750 for their VIS-NIR spectrum followed by the measurements of each property. 

The next step was to store fresh Barhi fruits in three systems: ambient (25 °C), cold (1 °C), and CA (1 °C with 5%:5% O_2_:CO_2_, 85% RH) storage, for up to three months. Typically, during storage, Barhi fruits tend to mature from the Khalal stage (yellow) to the Rutab stage (dark brown). The aim of proper Barhi storage is to delay such ripening to avoid losing the consumers’ preference when fruits become brown in color. In this study, the storage for date fruits would be terminated once the ripening to brown exceeded 50% of each batch of fruits.

### 2.2. Sensory Analysis

Fresh Barhi date fruits (80–100% yellowish) were sensory evaluated by thirty-six evaluators from King Saud University at the College of Food and Agricultural Sciences. To conduct the planned sensory evaluation, safety and health precautions were taken. The sensory evaluation process was accomplished using the 9-point hedonic table [27,28]. The selected sensory attributes were taste, texture, color, and general acceptance, with responses ranging from 1, denoting “extremely dislike”, to 9, denoting “extremely like”. The fruits were examined at 5 °C right after being precooled or taken from cold stores for safety issues.

### 2.3. Objective Analysis

A digital refractometer (Abbe 5 Refractometer, Bellingham, Stanley (BS), Jena, Germany) was used at room temperature (25 °C) to measure the TSS of Barhi fruits (expressed as a percentage) [29]. 

The color of the Barhi fruits was assessed using a Hunter Lab scan XE (Hunter Associates Laboratory Inc., Reston, VA, USA) and the fundamental color criteria L*, b*, and a*, where L* stands for brightness/darkness, a* stands for redness/greenness, and b* stands for blueness/yellowness. The browning index (BI) and total color difference (∆E) were derived from the L*, a*, and b* values [30] according to:

(1) $$\Delta E=\sqrt{{\left({{\;L}^{*}}_{0}\;-L^{*}\;\right)}^{2}+{\;\left(\;{a^{*}}_{0}\;-a^{*}\;\right)}^{2}+{\;\left({b^{*}}_{0}\;-\;{\;b}^{*}\;\right)}^{2}}$$

(2) $$\mathrm{BI}=\frac{\left[100\;\times \;\left(\;x\;-\;0.31\;\right)\right]}{0.170}$$

where

(3) $$x=\frac{\left(\;a^{*}+1.75L^{*}\;\right)}{\left(\;5.645L^{*}+a^{*}-\;3.012b^{*}\;\right)}$$

To evaluate the fruit texture, TA-hDi (Model HD_3128, Stable Micro Systems, Surrey, UK) texture analyzer was utilized. A compression cylinder (P 75) was used in the texture profile analysis test (TPA) to achieve a 5 mm deformation at 1.5 mm/s with an average diameter of 15 mm +/− 0.2. Using the resulting deformation curves of the force–time, the hardness parameter was derived [1]. To estimate the moisture content (MC%), the AOAC procedure was followed, and samples were dried under vacuum (200 mmHg) for 48 h at 70 °C (D_63450-VT 6025, Heraeus Inc., Hanauer, Germany) [18].

### 2.4. Evaluation of the Quality Index (Q_i_)

The Q_i_, which has a range of 0 to 1, is a tool used to model and normalize the variables under study in relation to the controlled variable’s minimal value. Normalizations help to ensure that the data in the Q_i_ are compatible [31]. The following formula can be implemented to normalize the parameters:

(4) $$\hat{x_{i}}=\frac{x_{i}-x_{min}}{x_{max}-x_{min}}$$

where $\hat{x_{i}}$ is defined as the normalized value of the quality parameter x, and $x_{min}\;and\;x_{max}$ are the minimum and maximum values of x, respectively. Calculation of the quality index (Q_i_) was according to:

(5) $$Q_{i}=\frac{\sum_{1=1}^{n}\hat{x_{i}}}{n}$$

where n is the number of samples. Consequently, normalized sample characteristics and overall sensory acceptability data were combined in the generated Q_i_.

### 2.5. VIS-NIR Measurements

Using a portable VIS-NIR spectrometer (F-750, Firmware v_1_2_0 build 7041, Felix Inst., Camas, WA, USA) with a wavelength range from 285 to 1200 nm, and then using F-750 Software (Data Viewer, version v1 1.0.51), the data from both measurements—reference values and absorbance—were uploaded and examined. The second derivative from the reflectance curve in the range of 285–1200 nm was then obtained using another piece of software (Model Builder (version v1 1.0.105), Zeiss MMS1 VIS-NIR spectrometer, Felix Inst., Camas, WA, USA) with an interval of 3 nm used to set the system. For every fruit sample, optical shots of the F-750 were obtained prior to food measurements (sensory evaluation and objective attributes). The F-750 has a reference shutter that, while scanning with the lamp off, makes it possible to account for the ambient light and dark current in each measurement. Three scans of each sample of the spectrum were captured and then averaged. Spectra were acquired for a different group of samples at 5 °C (similar to the sensory temperature) for the same serving for the validation procedure. After the spectra were recorded, all data on the F-750 SD card were transferred to a computer for further analysis. Using Data Viewer Software, the imported data were saved in CSV format, and the spectra were pre-analyzed utilizing the Savitzky–Golay second derivative.

### 2.6. Statistics and Analysis

Utilizing statistical software (SAS, V. 9.2, Cary, NC, USA), all objectively measured attributes were examined. Felix F750 AppBuilder v.2.1.7 software was used to predict the performance of both calibration and cross-validation findings. Microsoft Office 365 was used to create graphs, plots, and other calculations. A total of 1000 fruits were divided into three groups; each represented a stage of ripening for the calibration test (training set). Cross-validation was used to test the model once it was built.

Partial least squares regression (PLSR) and artificial neural network (ANN) analysis tools were used to generate the models of calibration. The spectra are examined as a linear multivariate connection using the PLSR method [32]. Prior to applying the multiple regression model, PLSR finds the high-dimensional vectors (latent variables, LVs) that are used to explain the data’s most valuable variation. Exploring subspaces that boost predictor and response variable covariance leads to the discovery of latent variables [33]. The highly developed nonlinear pattern recognition technique known as artificial neural networks (ANNs) can simulate complex biodiversity as well as instrument and environmental variability; ANNs are a prominent technology used in VIS-NIR spectroscopy as an alternative to PLS. Unlike PLS, ANNs can handle non-linearity and provide many optimization options. To detect spectral similarities, a large training dataset and discriminant-based methods were employed. Furthermore, ANNs can fit unknown spectra to numerous comparable spectra, whereas PLS can only multiply and add spectra from the calibration set. This makes ANNs more dependable when dealing with varied spectra [34]. In this study, App-Builder v.2.1.7 software (Felix Instr., Camas, WA, USA) was used to examine the estimated data. Based on calibration and validation results, the ANN performance of the prediction was evaluated using the correlation coefficient (R^2^), square error of root mean in calibration (RMSEC), and cross-validation (RMSECV) [14]. RMSEC is a parameter that indicates how well the calibration model coincides with the calibration set.

## 3. Results

Based on the Barhi date characteristics, the shelf life of the fruits was objectively and subjectively evaluated, and then the Q_i_ and VIS-NIR were used for modeling.

### 3.1. Sensory Evaluation

The average sensory evaluations of Barhi fruit characteristics by judges are displayed in Table 1, which shows the significant variations determined utilizing Duncan’s multiple-range test (*p* < 0.05).

Table 1 shows that the two most popular samples were 90% and 100% yellowish fruit. However, 90% yellowish dates are preferred compared to 100% yellowish dates to achieve a delay in ripening and prolong the shelf life of the stored fruits. This is because they have better texture and cohesion properties as well as crunchiness, are not susceptible to deterioration or an increase in ripeness, and do not turn into Rutab easily. Table 1 shows that the harvesting time (stage of maturity) had a significant influence (*p* < 0.05) for all sensory acceptance parameters in which 80% yellowish date fruits had the least liked taste, color, and overall acceptance. Therefore, the recommended 90% yellowish date fruits were selected for the shelf-life storage for up to 120 days (CA storage), 40 days (cold), and 20 days (ambient, marketing temperature) based on their physical and sensory characteristics.

#### Sensory Assessment of Fruits during Storage

Table 2 shows the average sensory attributes of the Barhi samples during the storage period of up to 120 days (CA storage), 40 days (cold) and 20 days (ambient conditions). The periods of storage were determined based on when 50% of the samples deteriorated. Deterioration was detected visually based on the transformation of Khalal (yellow) into Rutab (brown). During storage, the sensory scores for the qualitative attributes (taste, texture, color, and overall acceptance) for all samples decreased over time. This indicated that the Barhi fruits deteriorate with storage. At the end for each of the storage period, the color scores in CA, cold, and ambient decreased from 8.9 to 3.30, 3.45 and 3.52 while texture assessments decreased from 8.4 to 3.13, 3.06 and 3.20 where taste average scores decreased from 8.7 to 3.8, 4.05 and 3.89, respectively. Furthermore, the evaluations for “overall acceptance” decreased from 8.5 to 2.84, 3.83 and 3.70, respectively. It can be noted, however, that fruit quality was much better in CA storage compared to cold and ambient conditions for the 40- and 20-day storage periods, respectively.

### 3.2. Evaluation of Barhi Fruits Physical Properties during Storage

Table 3 shows the influence of storage time on the physical properties of Barhi fruits. The TSS% scores for CA, cold, and ambient (25 °C) increased from 20.31 to 24.12, 23.39, and 25.03%, respectively, at the end of storage, which reflects the loss of moisture during storage. ∆E increased from 0 (control, fresh fruit) to 11.68, 11.28, and 12.16 where the average BI score increased from 83.31 to 89.45, 91.45, and 89.76, for CA, cold, and ambient storage, respectively. Furthermore, the hardness decreased from 99.81 to 73.59, 76.85, and 52.29 N where MC% decreased from 70.99 to 62.64, 62.58, and 60.91% for CA, cold, and ambient storage, respectively. This reflects the gradual deterioration of fruits during storage but at different rates based on storage conditions.

### 3.3. Modeling of Quality Index (Q_i_)

**Figure 1:**
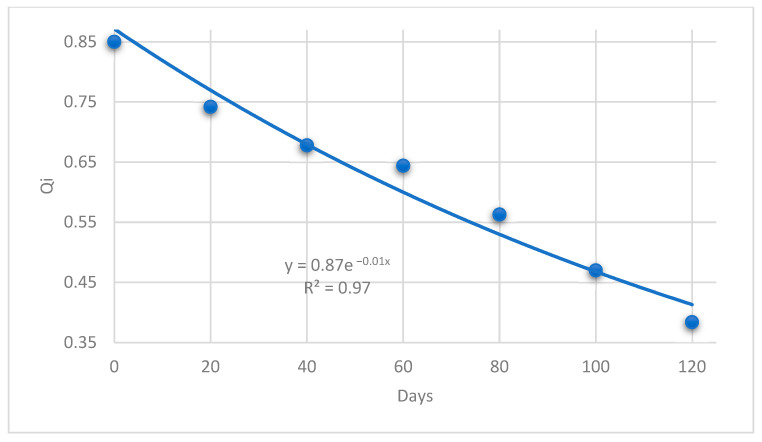
Normalized Q_i_ of Barhi fruits during controlled atmospheric storage.

The Q_i_ for Barhi evaluates the standard deviation of ten characteristics, five of which are sensorial and five of which are physical. The Barhi fruits displayed consistent and ongoing changes for all assessed parameters during the storage period, as presented in Figure 1. The Barhi fruit index of modeled quality began with a value of 0.85 and decreased to 0.38 at the end of the storage time.

The following model (with R^2^ = 0.97) established a relationship of the normalized Q_i_ and with duration of shelf-life (days). The power law model could be used to analyze a food product’s sensory evaluation [35]. Thus, the relationship between the quality index (Q_i_) and the shelf life is as follows:

Q_i_ = 0.87e^−0.01 Days^ (6)

Once the power function’s exponent approached less than 1.0, the overall acceptance exhibited a downward curve in relation to the normalized Q_i_ and rose more slowly as the Q_i_ increased.

It can be stated that the Q_i_ aided in providing a reference for the actual shelf life of Barhi fruits. Furthermore, authorities may utilize Q_i_ as a powerful tool to evaluate and determine the actual quality during the shelf life of Barhi fruits throughout the production, transportation, storage, and retail chain with tolerable precision.

### 3.4. Quality Index (Q_i_) Modeling with VIS-NIR Spectra

**Figure 2:**
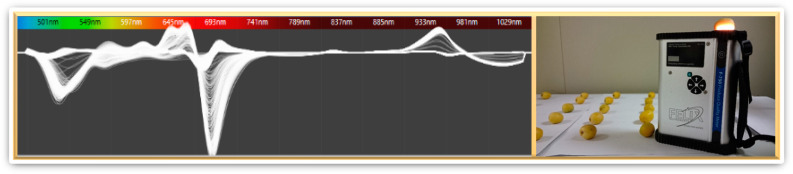
Spectra absorbance view of 1000 samples of Barhi dates at the different maturity levels utilizing the Flex F750 instrument.

VIS-NIR technology has recently acquired popularity in food applications for linking the spectrophotometer wave absorbance and reflectance with the physiochemical characteristics of fruit ingredients. To gain superior performance and powerful models, VIS-NIR spectrum data must be pre-processed. These derivatives can be quite beneficial in the near-infrared spectrum to eliminate some improper signals from the spectrum [36]. The second derivative of VIS-NIR spectra reflectance was obtained in this work). Figure 2 shows the spectra absorbance for Barhi dates (1000 fruits) for the different levels of fruit maturity (from 80 to 100% yellowish).

**Figure 3:**
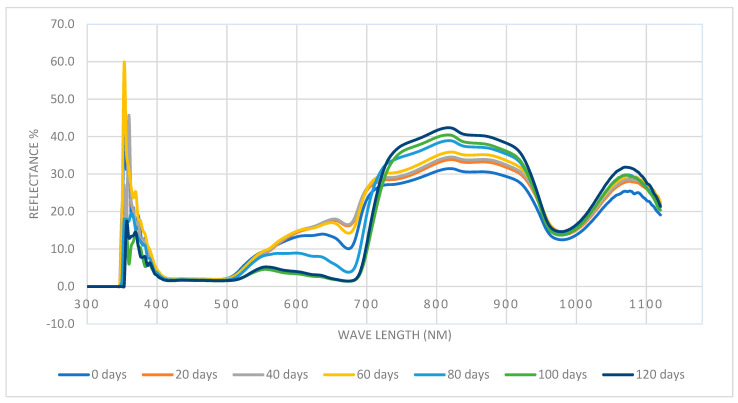
Reflectance spectra (%) at different storage periods in CA storage from fresh (0 days) to 120 days (n = 35 fruits for each storage period).

**Figure 4:**
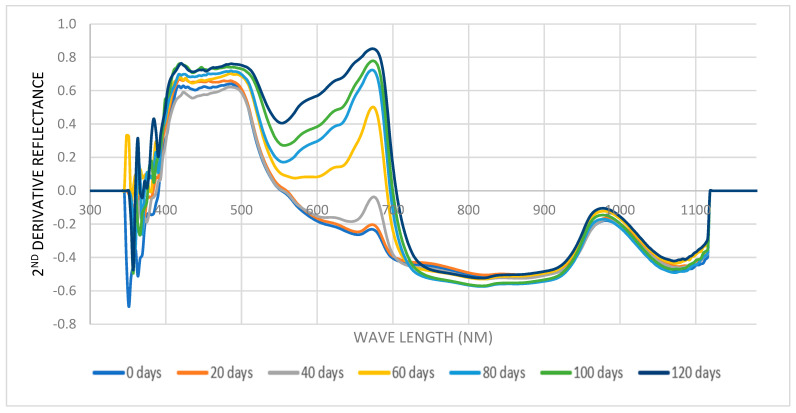
The second derivative reflectance mean of 250 Barhi date fruits at different storage periods from fresh (0 days) to 120 days.

The spectrum reflectance was typically in the visible region (from 475 to 650 nm) [37]. Figure 3 depicts the spectra (reflectance (R), %) with the longest storage span being 120 days; Figure 4 shows the average second derivative of Barhi fruit spectra reflectance at different storage periods along the storage span of 120 days. These figures demonstrate the effect of the storage period on the reflectance and second derivative curves at a wavelength 350 to 1100.

VIS-NIR spectra will be further analyzed utilizing two powerful tools, namely PLSR and ANN, and will then be tested for its performance for the quality of Barhi fruits (objective analysis) compared with Q_i_.

### 3.5. Partial Least Squares Regression (PLSR)

Table 4 presents calibration analysis and the cross-validation of PLSR for the Barhi fruits physical properties of TSS%, ∆E, BI, hardness (N), and MC% as well as Q_i_ characteristics. In the calibration models, R^2^ and RMSEC (mmd^−1^) were 0.979 and 0.659 for TSS%, 0.961 and 0.994 for ∆E, 0.881 and 0.978 for BI, 0.903 and 0.708 for hardness (N), 0.902, 2.119 for MC%, and 0.793 and 0.110 for Q_i_, respectively.

In cross-validation analysis, R^2^ and RMSECV were 0.910 and 0.758 for TSS%, 0.912 and 0.979 for ∆E, 0.882 and 0.902 for BI, 0.893 and 0.777 for hardness (N), 0.901 and 1.921 for MC%, and 0.783 and 0.298 for Q_i_, respectively. The coefficient of correlation (R^2^) data was in a range between 0.793 and 0.979. Such a range proves the high performance of the model, with an R^2^ greater than 0.70 regarded as satisfactory in NIR models [38,39]. This implies that the PLSR analysis is an effective statistical means for predicting both Q_i_ and objective Barhi fruit quality indicators.

### 3.6. Artificial Neural Networks Analysis (ANN)

The performance analysis of ANN for each calibration and cross-validation of the Barhi fruit characteristics is shown in Table 5. In the calibration models, R^2^ and RMSEC were 0.981 and 0.857 for TSS%, 0.950 and 1.093 for ∆E, 0.891 and 0.681 for BI, 0.891 and 0.747 for hardness (N), 0.901 and 1.82 for MC%, and 0.912 and 0.308 for Q_i_, respectively. Moreover, regarding the cross-validation performance, R^2^ and RMSECV were 0.979 and 0.705 for TSS%, 0.949 and 0.989 for ∆E, 0.889 and 0.605 for BI, 0.893 and 0.708 for hardness (N), 0.901 and 1.129 for MC%, and 0.912 and 0.308 for Q_i_, respectively. The results showed that the ANN analysis is a powerful tool for the estimation of Barhi date properties. It is interesting to note that compared to the PLSR model, the ANN model is typically better suited for predicting Barhi characteristics, as shown by the higher correlation coefficients of the ANN analysis.

With the increased number of components, RMSEC decreases. RMSECV, on the other hand, grows as more components are included. Compared to RMSEC, RMSECV is a more accurate predictor of future model performance [17]. Therefore, in terms of calibration and cross-validation, the two analysis methods (PLSR and ANN) can be useful tools for modeling VIS-NIR data.

### 3.7. Performance of Prediction Models of Barhi Quality during Storage

**Figure 5:**
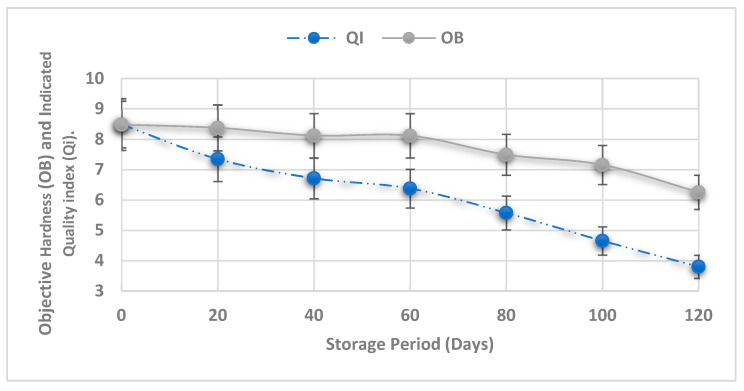
Indicated quality index (Q_i_) and objective hardness (OB) during CA storage (5%O_2_, 5%CO_2_, 80%RH). H: hardness × 1/11.76; Q_i_: quality index × 10 (n = 35 fruits for each storage period).

As indicated above, excellent model performance is achieved with R^2^ values greater than 0.70, in which values ranging from 0.50 to 0.69 indicated a fair model performance [40]. Figure 5 shows a comparison of sensorial data given by Q_i_ and objective TPA hardness tests. Both tests’ curves showed a similar trend in a decrease with storage time. This indicates that a link between the sensory and objective measurements utilizing Q_i_ and VIS-NIR spectra can be implemented in the industrial automation process of quality assurance.

**Figure 6:**
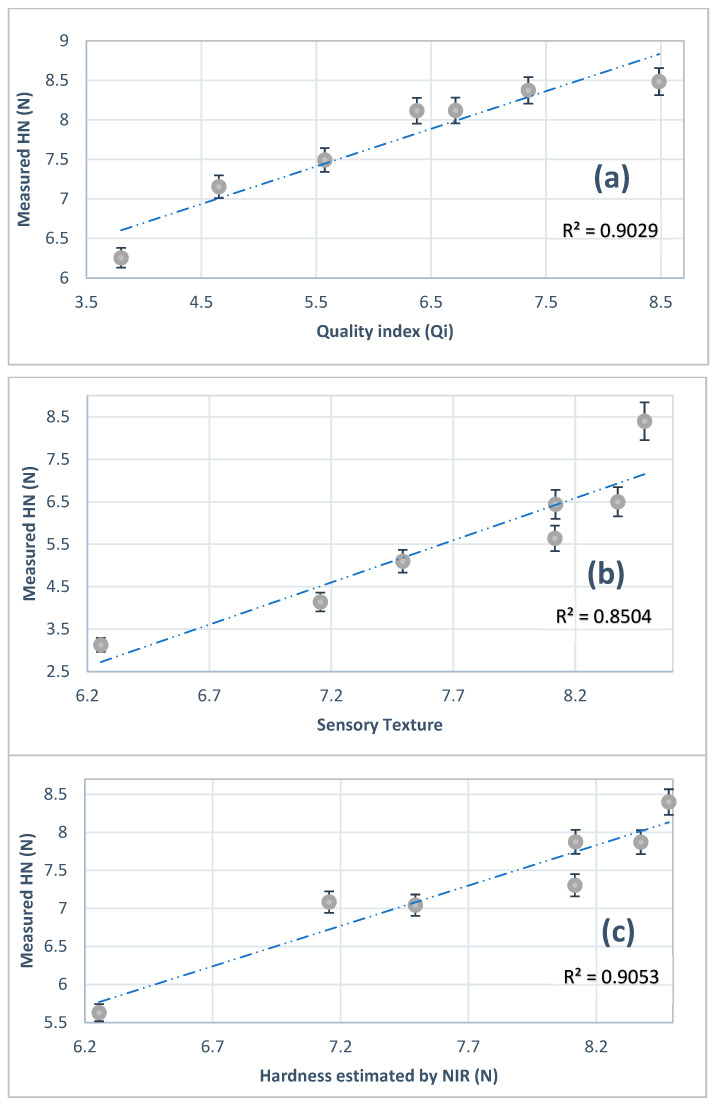
(**a**) Quality index Q_i_ and measured hardness, (**b**) sensory texture and measured hardness, and (**c**) relationship between hardness estimated using VIS-NIR with ANN and measured hardness (N). HN: hardness × 1/11.76 Q_i_: quality index × 10 (n = 35 fruits for each storage period).

Figure 6a shows the predicted Q_i_ and measured objective hardness. The correlation between these parameters was particularly good with a correlation coefficient of R^2^ of 0.902. Figure 6b shows a good relationship between sensorial and objective texture (hardness estimation) with a correlation coefficient of R^2^ = 0.850.

Figure 6c shows the relationship between VIS-NIR estimation and measured objective hardness with a correlation coefficient R^2^ = 0.905. This result proves how well VIS-NIR spectroscopy can correlate with textural measurements of Barhi fruits at different stages of maturity.

## 4. Discussion

According to the samples’ sensory analysis results, the acceptability of the arbitrators increases with fruit maturation. This is consistent with an increase in fruit maturity effect on sweetness (and, consequently, taste). These results agree with earlier studies on the influence of the harvesting stage on the storage duration of Barhi fruits [41] with ranges of 60 to 80% MC and 20 to 35% TSS, and yellow to green color based on sensory estimation, which affect consumer acceptance of Barhi fruits during the maturation process.

Preservation in CA storage achieved the longest shelf-life compared to cold and ambient storage based on Barhi fruit sensory assessment; CA storage could delay the deterioration up to 120 days. In an ambient environment, the samples had the shorted shelf-life. This could be attributed to the accelerated enzyme activities and texture softening at room temperature, which affect Barhi fruit quality during such storage [18].

The increase in TSS of fruits throughout the storage period is probably due to the loss of moisture and more polysaccharides being enzymatically converted to simple sugars [42]. The observed color variations and alterations may be related to differences in the enzymatic oxidation of phenolic chemicals. The cell structure of date flesh changes due to dehydration during storage and thus increases enzymatic oxidation of phenolic compounds [43]. Several studies [43,44,45] showed comparable results to this study. The reduction in hardness during storage may be related to cellular disintegration, which causes membrane porosity, or to the conversion of insoluble particles into soluble solids [46].

Regarding the VIS-NIR cross-validation performance, the R^2^ values were higher than 0.79, which indicates strong model performance, in which an R^2^ greater than 0.70 was regarded as satisfactory in VIS-NIR models [38,39]. The ANN model’s correlation coefficients are clearly greater than that of the PLSR model, especially when considering the normalized Q_i_ parameter. Therefore, it is advised that Q_i_ estimation would be dependent on the ANN technique (R^2^ = 0.891–0.981) rather than the PLSR technique (R^2^ = 0.792–0.979).

The quality index (Q_i_) of sensory and physical attributes simplified the assessment and comparison of product qualities, utilizing a non-destructive and simple VIS-NIR technique. As a result of the analyses in this work, VIS-NIR interrelated well with Q_i_ and, hence, with the Barhi fruit’s unique features. In general, Q_i_ can predict the fruit quality throughout the storage duration of Barhi dates and set by standard authorities [47]. Thus, the portable VIS-NIR spectra meter can be a helpful tool in the field for producers, manufacturers, storage, transportation, market, authorities, and throughout the entire chain to confirm the “quality” and “shelf life” of the commodities. The findings of this study indicate the feasibility of correlating non-destructive parameters (Q_i_ and VIS-NIR) with sensory estimation and texture parameters (hardness, N) of Barhi samples at any point during the storage time. This can be utilized in commercial online fruit sorting instrumentation using VIS-NIR instrumentation and the Q_i_ to predict the texture of fruit non-destructively.

Furthermore, this suggests that automated sorting of Barhi dates non-destructively based on fruit firmness is applicable using near-infrared spectroscopy. This can be utilized in the targeted markets; for example, fruit that is less mature and has a relatively hard texture might be assigned to long-distance/international markets, while relatively mature (mild texture) fruits may be assigned to intermediate-distance markets and soft fruits can be reserved for immediate marketing in regional/local stores. Moreover, based on VIS-NIR meters within the sorting line, fruits can be sorted automatically based on their “sweetness” and “moisture”. Additionally, VIS-NIR meters can be installed within fruit storage facilities to detect the quality and shelf life of fruits stored for weeks or months.

In essence, artificial intelligence (AI) can be integrated with near-infrared (VIS-NIR) spectroscopy to revolutionize fruit quality assessment. This powerful combination can predict ripeness, detect internal defects, and identify specific cultivars, enabling real-time monitoring and decision making throughout the food chain and optimizing postharvest management and processing for improved fruit quality with the highest possible efficiency.

## 5. Conclusions

The generated Barhi quality index (Q_i_) in this study provides an approximate estimation of fruit quality during its storage duration. This approach provides a quick, non-destructive, and reliable method for evaluating quality that is simple to apply across the entire chain of processing, storage, and marketing chains. The VIS-NIR analysis correlated the absorbance and reflectance of spectrophotometer waves to the physical characteristics of food products. During the shelf life, both sensory and objective measurements can be followed and evaluated with the quality index (Q_i_) and immediately measured using a VIS-NIR analyzer. Both (PLSR) partial least-square regression and (ANN) artificial neural network analyses were helpful to examine VIS-NIR validity.

VIS-NIR successfully predicted the Q_i_ and fits well with normalized measured sample properties (total soluble solids (TSS), color (∆E and Bi), texture (hardness), and moisture content (MC%)) and sensory evaluation during the shelf-life period. VIS-NIR spectroscopy was helpful in estimating the Q_i_ of Barhi fruits in calibration and cross-validation with an R^2^ range of 0.891–0.981.

In conclusion, VIS-NIR spectrophotometry can predict Q_i_ non-destructively and can thus be used successfully by authorities, product manufacturers, processors, and throughout the entire production, processing, transport, preservation, and retail market chain to assess the “quality” along the “shelf life” of the commodity.

## Figures and Tables

**Table 1:** Sensorial analysis of fresh Barhi fruits at several maturity levels * (n = 333 fruits for each ripening stage of maturity).

# Ripening Stage	Texture	Taste	Color	Overall Acceptance
1 (80% yellowish)	8.9 ± 0.41 ^a^	7 ± 0.08 ^b^	6.9 ± 1.06 ^b^	6.8 ± 1.08 ^b^
2 (90% yellowish)	8.4 ± 1.01 ^ab^	8.7 ± 0.86 ^a^	8.9 ± 0.49 ^a^	8.5 ± 0.38 ^a^
3 (100% yellowish)	8.2 ± 0.21 ^b^	8.8 ± 0.97 ^a^	8.8 ± 0.69 ^a^	8.5 ± 0.44 ^a^

* Average values within a column with same letters are not significantly different at *p* < 0.05.

**Table 2:** Variance analysis for the effect of storage period on the sensory assessment of Barhi fruits *^,^** (n = 36 fruits for each storage period/condition).

Days	Storage	Texture	Taste	Color	Overall Acceptance
0	CA, Cold, 25 °C	8.4 ± 1.01 ^a^	8.7 ± 0.86 ^a^	8.9 ± 0.49 ^a^	8.5 ± 0.58 ^a^
20	CA	7.02 ± 0.61 ^a^	7.5 ± 0.54 ^a^	7.12 ±0.05 ^ab^	7.42 ± 0.79 ^ab^
	Cold	6.52 ± 0.41 ^ab^	6.74 ± 0.93 ^abc^	6.94 ± 0.52 ^ab^	6.64 ± 0.39 ^abc^
	25 °C	3.20 ± 0.64 ^de^	3.89 ± 0.71 ^de^	3.52 ± 0.29 ^e^	3.70 ± 0.43 ^e^
40	CA	6.44 ± 0.81 ^ab^	6.89 ± 1.02 ^ab^	6.31 ± 0.25 ^bc^	6.78 ± 0.27 ^ab^
	Cold	3.06 ± 1.04 ^e^	4.05 ± 0.13 ^de^	3.45 ± 0.69 ^e^	3.83 ± 0.57 ^de^
	25 °C	N/A	N/A	N/A	N/A
60	CA	5.64 ± 0.97 ^bc^	6.31 ± 0.52 ^bc^	5.53 ± 1.12 ^cd^	6.44 ± 0.87 ^bc^
	Cold	N/A	N/A	N/A	N/A
	25 °C	N/A	N/A	N/A	N/A
80	CA	5.1 ± 0.93 ^c^	5.70 ± 1.62 ^c^	4.87 ± 1.03 ^d^	5.63 ± 1.62 ^c^
	Cold	N/A	N/A	N/A	N/A
	25 °C	N/A	N/A	N/A	N/A
100	CA	4.14 ± 1.07 ^d^	4.79 ± 0.71 ^d^	3.93 ± 0.94 ^e^	4.70 ± 0.92 ^d^
	Cold	N/A	N/A	N/A	N/A
	25 °C	N/A	N/A	N/A	N/A
120	CA	3.13 ± 0.79 ^e^	3.80 ± 0.09 ^e^	3.30 ± 0.82 ^e^	3.84 ± 0.19 ^de^
	Cold	N/A	N/A	N/A	N/A
	25 °C	N/A	N/A	N/A	N/A

* Average values within a column of a group with same letters are not significantly different at *p* < 0.05. ** (N/A): not applicable due to fruit deterioration (50% or more).

**Table 3:** Variance analysis for the influence of storage duration on the objective characteristics of Barhi fruits *^,^**. (n = 12 fruits for each measurement during various storage periods and conditions).

Days	Storage	TSS%	∆E	BI	Hardness (N)	MC %
0	CA, Cold, 25 °C	20.31 ± 1.08 ^d^	0 ^g^	83.31 ± 0.64 ^e^	99.81 ± 0.06 ^a^	70.99 ± 0.36 ^a^
20	CA	20.46 ± 0.35 ^d^	6.80 ± 0.79 ^f^	83.33 ± 0.06 ^e^	98.52 ± 0.63 ^a^	68.35 ± 0.91 ^b^
	Cold	21.56 ± 1.21 ^d^	9.06 ± 0.02 ^e^	87.21 ± 0.39 cd	83.65 ± 1.20 ^d^	65.25 ± 0.89 ^d^
	25 °C	25.03 ± 1.26 ^a^	12.16 ± 0.06 ^a^	89.76 ± 0.63 ^b^	52.29 ± 1.64 ^g^	60.91 ± 0.94 ^g^
40	CA	20.69 ± 0.86 ^d^	9.36 ± 0.08 ^de^	83.71 ± 0.16 ^e^	95.52 ± 0.43 ^b^	66.27 ± 0.49 ^c^
	Cold	23.39 ± 1.13 ^bc^	11.28 ± 0.04 ^bc^	91.45 ± 0.67 ^a^	76.85 ± 0.92 ^e^	62.58 ± 0.39 ^f^
	25 °C	N/A	N/A	N/A	N/A	N/A
60	CA	21.22 ± 0.73 ^d^	9.95 ± 0.82 ^d^	84.60 ± 0.67 ^e^	95.49 ± 0.92 ^b^	65.72 ± 0.71 ^cd^
	Cold	N/A	N/A	N/A	N/A	N/A
	25 °C	N/A	N/A	N/A	N/A	N/A
80	CA	21.58 ± 1.06 ^d^	10.93 ± 0.49 ^c^	86.21 ± 0.92 ^d^	88.15 ± 0.26 ^c^	65.35 ± 0.84 ^d^
	Cold	N/A	N/A	N/A	N/A	N/A
	25 °C	N/A	N/A	N/A	N/A	N/A
100	CA	22.64 ± 0.96 ^c^	11.48 ± 0.63 ^b^	87.94 ± 0.08 ^c^	84.18 ± 0.07 ^d^	64.13 ± 0.53 ^e^
	Cold	N/A	N/A	N/A	N/A	N/A
	25 °C	N/A	N/A	N/A	N/A	N/A
120	CA	24.12 ± 1.16 ^ab^	11.68 ± 0.37 ^ab^	89.45 ± 0.83 ^b^	73.59 ± 1.21 ^f^	62.64 ± 0.19 ^f^
	Cold	N/A	N/A	N/A	N/A	N/A
	25 °C	N/A	N/A	N/A	N/A	N/A

* Average values within a column of a group with same letters are not significantly different at *p* < 0.05. ** (N/A): not applicable due to fruit conversion from Khala (yellow) to Rutab (brown) (50% or more).

**Table 4:** Performance of PLSR for Barhi fruits for both calibration and cross-validation models for TSS%, ∆E, BI, hardness (N), MC%, and Q_i_ (n = 187 fruits for each property).

Parameter	Calibration		Cross-Validation	
	R^2^	RMSEC	R^2^	RMSECV
TSS%	0.979	0.659	0.910	0.758
∆E	0.961	0.994	0.912	0.979
BI	0.881	0.978	0.882	0.902
Hardness (N)	0.903	0.708	0.893	0.777
MC %	0.902	2.119	0.901	1.921
Q_i_	0.793	0.110	0.783	0.298

**Table 5:** The model performance of ANNs for both calibration and cross-validation for ∆E, TSS, BI, hardness, MC, and Q_i_ of Barhi fruits (n = 187 fruits for each property).

Parameter,	Calibration		Cross-Validation	
	R^2^	RMSEC	R^2^	RMSECV
TSS%	0.981	0.857	0.979	0.705
∆E	0.950	1.093	0.949	0.989
BI	0.891	0.681	0.889	0.605
Hardness (N)	0.891	0.747	0.893	0.708
MC%	0.901	1.822	0.901	1.129
Q_i_	0.912	0.308	0.912	0.308

## Data Availability

The data presented in this study are available on request from the corresponding author. The data are not publicly available due to privacy restrictions.
